# Prevalence and Characterization of Specific Phobia Disorder in People over 65 Years Old in a Madrid Community Sample (Spain) and its Relationship to Quality of Life

**DOI:** 10.3390/ijerph17061915

**Published:** 2020-03-15

**Authors:** Berta Ausín, Manuel Muñoz, Miguel Ángel Castellanos, Sara García

**Affiliations:** School of Psychology, Complutense University of Madrid, 28223 Campus de Somosaguas, Spain

**Keywords:** specific phobia disorder, elderly people, prevalence, level of functioning, quality of life

## Abstract

The prevalence of anxiety disorders over the last year among seniors ranged from 3.6% to 17.2%. The most prevalent disorders are specific phobias. Data are needed concerning the consequences of specific phobia disorder on the level of functioning and quality of life of older people, the age of onset of specific phobia disorder, and the duration of episodes. In total, 555 community-dwelling people aged between 65 and 84 years who lived in Madrid (Spain) were assessed (Composite International Diagnostic Interview for people over 65 years (CIDI65+), WHO Disability Assessment Schedule (WHODAS II), Health of the Nation Outcome Scales for Older Adults (HoNOS65+), World Health Organization Quality of Life Brief (WHOQOL-BREF). Prevalence rates and odds ratio, t-tests, binary logistic regression, and point-biserial correlations were calculated. A total of 12.07% of the sample suffered a specific phobia disorder over the last year. The average age at onset of the specific phobia was 38.78 (sd = 21.61) years. The mean duration of the phobia was approximately 20 (sd = 20) years. A significant effect of the specific phobia was found for the current levels of functioning and quality of life: WHOQOL-BREF total score (*p* < 0.05), WHODAS II overall score (*p* < 0.01), and HoNOS65+ total score (*p* < 0.001). Having specific phobia disorder decreased the level of functioning and negatively affected the quality of life. These data suggest the need for primary healthcare professionals to include the detection of specific phobia disorders in their protocols because people do not receive treatment for this problem, and they might carry it throughout their lives.

## 1. Introduction

Over the last 15 years, several studies and international organizations have highlighted the importance of mental health among the elderly [1,2,3,4,5,6]. The data concerning the prevalence of mental disorders among those over 65 years old are contradictory, as indicated by a meta-analysis conducted by Volkert, Schulz, and Härter et al. [7] in Western countries. The studies regarding anxiety disorders included in this meta-analysis show varying results with regard to prevalence, ranging between 2.3% and 8.9% [8,9,10,11]. The most prevalent disorders were specific phobias (current prevalence: 4.52%; lifetime prevalence: 6.66%). Similarly, a recent systematic review of the prevalence of anxiety disorders among people over 65 [12] found great variability in the results of 36 studies analyzed over the last 20 years. The prevalence of anxiety disorder over the last year among seniors ranged from 3.6% [3] to 17.2% [13]. This review [12], found that women are at a higher risk of suffering from an anxiety disorder over the last year than men [11,14,15,16,17,18]. In addition, the vast majority of studies found a negative correlation between age and the prevalence of an anxiety disorder [19,20], among others. The results regarding age and gender are in line with the most recently published study on the prevalence of anxiety disorders among the elderly [15]. Importantly, this review found that only three of the 36 studies reviewed exclusively analyzed the prevalence of anxiety disorders [14,19,21]. The remaining studies analyzed the prevalence of any mental disorder among the elderly. As such, most of the studies collected in this review analyzed the prevalence of anxiety disorders in general; that is, they did not analyze the prevalence differentiated by type of anxiety disorder. The disorders that were studied less frequently were agoraphobia [22], panic disorder [23], and generalized anxiety disorder [24]. To this end, we add that none of the studies included in Cisneros and Ausín [12] analyzed age of onset or the duration of anxiety episodes among people over 65 years old, nor did it describe the symptomatology of this disorder.

With respect to the method of evaluating anxiety disorders,12 studies included in the review [12] used the Composite International Diagnostic Interview (CIDI) of the World Health Organization [25], and four studies applied a variant of the Mini-International Neuropsychiatric Interview (MINI [26]). Another four studies used the computer-assisted Étude sur la Santé des Aînés Diagnostic Questionnaire (ESA-Q [27]).

To complement the results presented thus far, it is necessary to cite the latest epidemiological study focused on the mental health of people over 65 years old in Europe, the MentDis_ICF65+ study [13,15,28]. This study is the first to use an instrument adapted for seniors: the Composite International Diagnostic Interview for older people; a standardized and structured interview for the diagnosis of mental disorders based on the characteristics of older people (CIDI65+ [29]). Andreas et al. [13] found a 12-month prevalence of anxiety disorders in Europe of 17.2%; thus, anxiety disorders are the most prevalent mental disorder. The same study found a 12-month prevalence of specific phobia of 9.2% [13]. Becker et al. [30] found that specific phobia is a common mental disorder with a cross-national lifetime prevalence of 7.4%. In the study by Wardenaar et al. [31], the cross-national lifetime and 12-month prevalence rates of specific phobia were, respectively, 7.4% and 5.5%, and was higher in females (9.8% and 7.7%) than in males (4.9% and 3.3%) and higher in high/higher-middle income countries than in low/lower-middle income countries. The median age of onset was young (8 years). The study conducted by Sancassiani et al. [32], which includes in the sample only 8 persons over 65, shows a lifetime prevalence of the specific phobia of 2.3%. Females showed more than twice the frequency of males (*p* < 0.0001). These gender differences in the prevalence of specific phobia are described in different studies, although in all of them the older population is poorly represented [31,32,33,34,35,36,37]. Additionally, in all of the above studies, prevalence rates have been shown to decrease with age.

Although the studies mentioned above have illuminated the prevalence of specific phobia among the elderly, studies describing the symptoms of specific phobia disorder among these people and their differences based on age and gender are lacking.

At the same time, if attention is focused on the effect that anxiety disorders have on the functioning and quality of life of older people, then relatively little data are found about the general effects of suffering from an anxiety disorder. Likewise, the few studies published found that anxiety disorders worsen the level of functioning and quality of life of people over 65 years old [15,38,39,40,41,42,43]. Based on our searches, the data regarding specific phobia disorder are very limited in the older population. The few studies that find a decrease in quality of life and level of functioning in people with specific phobia include few older people in the sample and almost no people over 75 [31,32,37,38,39,40]. Therefore, data are needed concerning the consequences of specific phobia disorder on the level of functioning and quality of life of older people; moreover, studies that illuminate the age of onset of specific phobia disorder and the duration of episodes among older people are needed.

Because of its high prevalence, lifetime persistence, associated impairment, and high lifetime comorbidity rate with other disorders (with estimated rates of up to 81.0%), specific phobia is important from both an epidemiological and a clinical perspective [31].

Accordingly, the current study has four main objectives. First, we analyzed the 12-month prevalence of specific phobia disorder among people over 65 years of age in Madrid and the differences based on age and gender. This information will enable health service planners to have approximate data on the population of people over 65 in Madrid who need care for their problems arising from specific phobia. Second, we analyzed the age at onset of the first episode of specific phobia in this population and the duration of the episodes suffered throughout life. Third, we described the types of phobic stimuli and the symptomatology shown by participants across the four types of specific phobia disorder (animals, natural environments, blood/injection/injury, and situational). Finally, we analyzed the effect of specific phobia disorder on the level of functioning and quality of life of participants.

## 2. Materials and Methods

### 2.1. Sample and Procedure

The sample was obtained from the MentDis_ICF65+ Study, regarding the health and well-being of people between 65 and 84 years in Europe. This longitudinal study was conducted across six European cities [28]. The sample was randomly selected from the population over 65 years but younger than 84 years old in the Community of Madrid (Spain). The 21 districts of Madrid were included, and a random sample of the rural areas was conducted. A total of 555 people who met the inclusion criteria were interviewed.

The inclusion criteria of the sample were as follows:

- Living in Madrid;
- Between 65 and 84 years of age;
- Able to provide informed consent to participate in the study.

The exclusion criteria for the sample were as follows:

- Presenting with a severe cognitive impairment as evaluated using a Mini-Mental State Examination [44] cut-off point of > 18;
- Having a language barrier that prevented an interview.

Informed consent was requested from the people in the sample, and all procedures and strategies used in the study were approved by the Ethical Committee of the Complutense University of Madrid (Universidad Complutense de Madrid) and the European Commission. 

Table 1 shows the sociodemographic characteristics of the sample. The mean age was 73.5 years.

### 2.2. Instruments

#### 2.2.1. Evaluation of Specific Phobia Disorder

To evaluate and diagnose specific phobia disorder, the Composite International Diagnostic Interview for people over 65 years (CIDI65+) was applied [29]. This standardized diagnostic interview was used to collect the lifetime, 12-month, and current prevalence data of mental disorders among elderly people. The interview evaluates the main disorders included in the Diagnostic and Statistical Manual of Mental Disorders-Revised (DSM-IV-TR). Thus, the CIDI65+ yields diagnoses based on the criteria of the DSM-IV-TR classification system [45]. In addition to diagnosis, it provides information about the types of specific phobia (i.e., animals, natural environments, blood/injection/injury, and situational) and their symptomatology. The test-retest reliability of this interview is acceptable for anxiety disorder (κ = 0.62, range = 0.30–0.78; [29]). The CIDI65+ interview includes questions about the onset and recency of each specific phobia episode the evaluated person suffered.

The types of specific phobia contemplated by the DSM-IV-TR and the DSM-5 [46] are the same. The diagnostic criteria of DSM-5 for this disorder are 7 criteria, as in DSM-IV-TR. Differences are found regarding the order of the criteria but the modifications are minimal in their content, although the wording is not exact. The DSM-5 broadens the criterion that fear, anxiety, or avoidance typically lasts 6 or more months, also in those over 18 years.

#### 2.2.2. Level of Performance Evaluation

The WHO Disability Assessment Schedule (WHODAS II [47]) was used to measure health and disability based on the International Classification of Functioning, Disability, and Health (ICF [48]). The version used in this study consists of 12 items, in which the person evaluated reports the difficulties that he or she has had over the last month in performing different activities and common tasks (i.e., moving, taking care of household responsibilities, social and cognitive functioning, self-care, and participation). The answers ranged from 1 (“no difficulty”) to 5 (“extreme difficulty/cannot do”). This study applied the overall score. The test-retest reliability of this instrument was 0.98, and the internal consistency was 0.98.

To complement the survey above, the Health of the Nation Outcome Scales for Older Adults (HoNOS65+) was used [49,50]. This measure consists of 12 scales that assess different aspects of health and psychosocial functioning that the interviewer records based on all of the available information about the person being evaluated. The responses to the 12 scales that compose it are scored from 0 to 4, where 0 is “without problem” and 4 is “serious problems” in that area. On this occasion, the total score was used. The Spanish adaptation of the HoNOS65+ [39] has an internal consistency of 0.65. The Pearson’s correlation values between most of the HoNOS65+ scales and the other tests evaluating similar constructs are significant.

#### 2.2.3. Evaluation of Quality of Life

Quality of life was evaluated with the World Health Organization Quality of Life Brief (WHOQOL-BREF) [51]. This scale consists of eight items with five Likert-type response options. The person evaluated responds to different aspects related to the quality of their life over the last month. This scale also includes two additional overall questions: The items are grouped into three subscales (physical health, social relationships, and environment) and an overall score. In this case, the overall score was used. The WHOQOL-BREF has an internal consistency of >0.7 across the different scales. A factor analysis yielded four factors that explain 53% of the total variance.

### 2.3. Statistical Analyses

Calculations of the prevalence rates and odds ratios (ORs) were performed using the sample frequencies weighted by gender and age. To this end, population data from the INE (Instituto Nacional de Estadística—Statistics National Institute) [52] were used. The results in the tables include the 95% confidence intervals for the ORs and a contrast for the equality of odds between the comparison groups.

To analyze the age of onset of specific phobias, the data of 52 participants, who indicated that they had suffered from a specific phobia episode at some point in their life, were used. A total of 27 of these participants also indicated the age at which the phobia had ended (i.e., age of recency). Both variables, along with duration (i.e., the difference between the two), were described and checked for gender differences via t-tests. If the results did not meet the assumption of homoscedasticity, then the degrees of freedom were adjusted.

An analysis of the contribution of psychosocial factors was conducted using a binary logistic regression, in which the main variables recorded in the study (i.e., age, gender, and number of significant people in their lives) were used to predict specific phobia disorder (yes/no). The resulting model was constructed based on regression comparisons of nested models (χ^2^) and Wald contrasts. The McFadden pseudo-R^2^ and the Homer–Lemeshow goodness-of-fit tests are provided as indicators of fit.

To determine the influence of specific phobias on the level of functioning and quality of life, point-biserial correlations were calculated. Because the relationships between these variables and gender and age are well known, they were calculated as partial correlations (i.e., the correlation between the residuals of the regression lines for gender and age against level of functioning and quality of life).

R [53] was used for all analyses. The epitool package was used for the prevalence and OR analyses [54].

## 3. Results 

### 3.1. Twelve-month Prevalence of Specific Phobia Disorder

The results indicate that of the 555 people evaluated, 67 (12.07%) met the DSM-IV-TR diagnostic criteria for specific phobia over the last year. Table 2 shows the significant prevalence and OR values for gender and age. Women were more than twice as likely to suffer from a specific phobia disorder than men over the last year (OR_men/women_ = 0.38, 95% CIs = 0.21–0.69). The prevalence of specific phobia disorder decreased with age (OR_65–74/75–84_ = 2.08, 95% CIs = 1.18–3.79).

Table 2 also shows the lifetime, 12-month, and current prevalence data by type of specific phobia (i.e., animals, natural environments, blood/injection/injury, situational) and the ORs for gender and age.

Of the 67 people who met the DSM-IV-TR diagnostic criteria of specific phobia over the last year, some suffered from more than one type of specific phobia. Table 2 shows that 23 people suffered from animal phobias, 27 people had phobias of an aspect of the natural environment, 19 had phobias of blood/injection/injuries, and 14 had phobias of some situational aspect. Table 2 shows the prevalence and OR values associated with gender and age for each type of specific phobia. Women were seven times more likely to suffer from an animal-type specific phobia than men over the last year (OR_men/women_ = 0.1, 95% CIs = 0.01–0.4); this difference was significant. Women were twice as likely to suffer from a specific phobia of the natural environment over the last year than men (OR_men/women_ = 0.36, 95% CIs = 0.13–0.91), which was significant. Although women were more likely to suffer from a blood/injection/injury specific phobia over the last 12 months, this value was not significant (OR_men/women_ = 0.62, 95% CIs = 0.2–1.74). Finally, women were more likely to suffer from situational-type specific phobia, although the values were not significant (OR_men/women_ = 0.42, 95% CIs = 0.1–1.49). This same trend was observed for lifetime and current prevalence (Table 2).

Table 2 shows that the 12-month prevalence of all types of specific phobias decreased with age and phobias of some aspect of the natural environment were significant. The OR (95% CI) values associated with the phobias regarding animals, some aspect of the natural environment, blood/injection/injury, and situational aspects were 1.38 (0.54–3.68), 2.60 (1.04–7.42), 1.21 (0.44–3.53), and 0.65 (0.18–2.17), respectively. The same trend was observed for lifetime and current prevalence (Table 2).

To analyze the contribution of psychosocial factors (i.e., age, gender, and number of significant people) with regard to the appearance of specific phobia disorder, a binary logistic regression was conducted. The McFadden pseudo-R^2^ value was 0.07, and the Hosmer–Lemeshow goodness-of-fit test was not significant (χ^2^ = 11.50; df = 8; *p* = 0.175). Table 3 shows that the coefficients obtained for each variable significantly differed from 0. This finding corroborates the significant differences between men and women (Table 2). Similarly, older participants were less likely to suffer from specific phobia disorder. Having more significant people in one’s life was a protective factor against suffering from specific phobia disorder among people over 65 years old.

### 3.2. Age of Onset and Duration of Specific Phobia Disorder

The average age at the onset of the specific phobia was 38.78 (sd = 21.61) years; although the age of onset for men was higher than that for women (42.26 and 36.78 years, respectively), This difference was not significant, t(50) = 0.87; *p* = 0.384. Of the 52 people who indicated the age of onset of the phobia, 25 also indicated the age of termination. An equivalent analysis of these 25 people using age of termination provided similar results. The mean age of recency (i.e., when the person says the specific phobia disorder has ended) was 57.48 (sd = 15.78) years; although a difference was found between women and men (53.58 and 64.10 years, respectively), it was not significant, t(25) = 1.73; *p* = 0.095. The mean duration of the phobia was 19.73 (sd = 20.48) years; although the duration was greater for men than for women (25.20 and 16.31 years, respectively), the groups were not different, t(24) = 1.07; *p* = 0.291.

**Figure 1:**
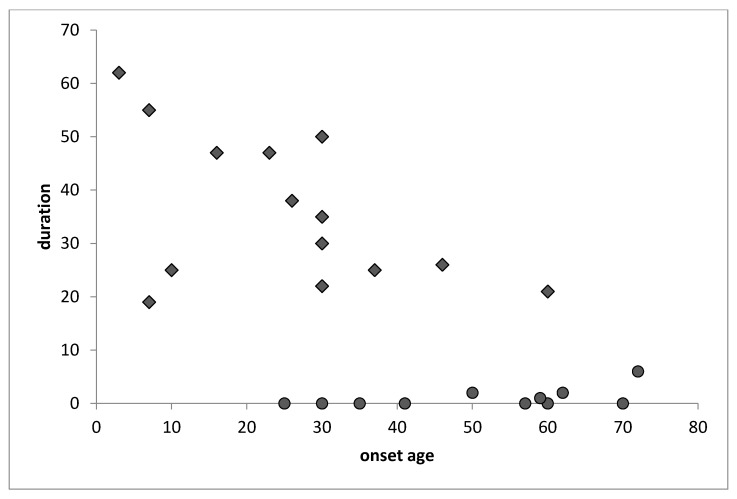
Relation between onset age and duration of the specific phobia disorder.

The relationship between the age of onset and the duration of the phobia was significantly correlated, r = −0.69; t(24) = 4.71, *p* < 0.001. Moreover, two clearly differentiated groups were observed (Figure 1): One group where the episode was punctual (i.e., they suffered from a single episode of specific phobia over their lifetime), regardless of the age at onset; and a second group where the phobia episode was briefer and appeared at an older age.

### 3.3. Description of the Symptomatology of the Specific Phobia Disorder

Table 4 shows the 12-month prevalence values, the ORs for gender and age, and the contrasts with OR = 1, with regard to problematic situations that generate anxiety responses in people with specific phobia disorder, as well as phobic stimuli and general symptomatology.

#### 3.3.1. Description of Problematic Situations

The types of problematic situations included in the analysis are shown in Table 4. The problematic situation that generated the most anxiety in the current sample was being in a crowd or in a line of people (43 people); the least anxiety-inducing situation was being in a public place, such as a store, market, theater, or parking lot. Differences were found between men and women in terms of situations that generated anxiety, although these differences were only significant in the case of “traveling alone or taking a long trip”; this situation was four times more likely to generate anxiety in women than men (OR_men/women_ = 0.23, 95% CIs = 0.02–1.11).

Table 4 shows that some situations were more likely to generate anxiety with age (e.g., the oldest people in the sample were almost twice as likely to fear “traveling alone or taking a long trip”, OR_65–74/75–84_ = 0.47, 95% CIs = 0.14–1.55), and other situations were likely to do so (e.g., older people had less fear of “traveling by bus, train, metro, or other public transportation”, OR_65–74/75–84_ = 1.30, 95% CIs = 0.44–4.03). No significant differences were observed based on age regarding any of the problematic situations studied.

#### 3.3.2. Description of Phobic Stimuli

The presence of the phobic stimuli included in the DSM-IV-TR was analyzed with regard to the sample and shown in Table 4. Of the 555 people in the sample, 285 did not report any fear of the stimuli indicated. The stimuli that generated the greatest anxiety responses were (in the following order) living things, such as insects, snakes, birds, or other animals; heights; enclosed spaces, such as caves, tunnels, or elevators; going to the dentist or a hospital; thunderstorms, thunder, or lightning; flying; seeing blood; getting an injection; and being in calm waters, such as a swimming pool or lake. Significant gender differences were found with regard to the probability of a type of stimulus triggering an anxiety response. Table 4 shows that these differences, where women were at the greater risk of suffering, were significant for the following stimuli: living things such as insects, snakes, birds, or other animals (OR_men/women_ = 0.25, 95% CIs = 0.16–0.39); thunderstorms, thunder, or lightning (OR_men/women_ = 0.25, 95% CIs = 0.11–0.51); being in calm waters, such as a swimming pool or lake (OR_men/women_ = 0.24, 95% CIs = 0.06–0.76); and enclosed spaces such as caves, tunnels, or elevators (OR_men/women_ = 0.36, 95% CIs = 0.21–0.61). Men were more likely to report not having any fear, and this difference was significant (OR_men/women_ = 2.07, 95% CIs = 1.45–2.95).

Age increased the probability of not having a phobia (OR_65–74/75–84_ = 0.56, 95% CIs = 0.39–0.79), and this effect was significant. Table 4 shows the significant differences in some of the stimuli studied by age. Specifically, younger people were less likely to fear living things, such as insects, snakes, birds, or other animals (OR_65–74/75–84_ 1.84, 95% CIs = 1.22–2.79); however, older people were less likely to fear thunderstorms, thunder, or lightning (OR_65–74/75–84_ = 1.97, 95% CIs = 1.05–3.84).

#### 3.3.3. Description of the Symptoms of Specific Phobia Disorder

The symptoms of specific phobia analyzed among the current sample were those included in the DSM-IV-TR and listed in Table 4. Of the people with a specific phobia in the last year, the most frequent symptoms were a stronger or faster heart beat (38 people), dry mouth (29 people), shortness of breath (28 people), and feeling suffocated (26 people). No significant differences were found between men and women or by age group regarding the symptomatologies presented (although 75- to 84-year-olds were at a lower risk of suffering from symptoms than their younger counterparts; Table 4).

### 3.4. Level of Functioning and Quality of Life

The regressions, including gender, age, and specific phobias, over the last year, with regard to the current levels of functioning and quality of life, revealed a significant effect for the WHOQOL-BREF total score (*p* < 0.05), the WHODAS II overall score (*p* < 0.01), and the HoNOS65+ total score (*p* < 0.001). Gender significantly predicted quality measurements (all *p* < 0.001); however, age was only significant for the WHODAS II overall and HoNOS65+ total scores (*p* < 0.001) and not for the WHOQOL-BREF total score (*p* = 0.320). The correlations (adjusted for gender and age) between specific phobia and quality of life are shown in Table 5. Although the correlations were significant (likely because of the sample size), the effect was small; the highest value was for the HoNOS65+ total score (r = 0.19), and little of the variances of level of functioning and quality of life were explained (ranging from 3% of the HoNOS65+ total score to 1% each of the WHOQOL-BREF total and WHODAS II overall scores).

Table 5 shows the point-biserial correlations adjusted by gender and age, as well as the relationships between specific phobia disorder and the WHODAS II overall, HoNOS65+ total, and WHOQOL-BREF total scores.

## 4. Discussion

The results show a 12-month prevalence of specific phobia disorder of approximately 12% among people over 65 years old. Once again, specific phobia was the most prevalent anxiety disorder. Thus, this study found a higher prevalence than all other previous studies of this age group, which is approximately 4.5% on average [7]. The use of adapted diagnostic tools and age-adjusted sampling systems might be the basis of this difference. One exception is the work of Andreas et al. [13], who found a 12-month prevalence of 9.2%. In that case, the instruments and methodology were the same, but the sample of the present study was a part of the MentDis_ICF65+ study; thus, the disparity should be explained as a function of differential prevalence across cities.

When analyzing the data by gender and age, we found that women were more than twice as likely to suffer from a specific phobia disorder than men; furthermore, its prevalence decreased in both genders with age. These data corroborate those found by Cisneros and Ausín [12], who analyzed the differences in the risk of suffering from an anxiety disorder and found an increased risk for women [11,14,15,17,18,27]. Likewise, differences were found with regard to the risk of suffering from an anxiety disorder as a function of age, with younger people (65–75 years) presenting with the greatest risk [19,20]. These results highlight the importance of considering gender and age when studying specific anxiety disorders in people over 65 years old [15]. Likewise, these differences are relevant to the identification of disorders and the personalization of treatments offered to this population in healthcare services.

Regarding the prevalence of the different types of specific phobia (i.e., animals, natural environments, blood/injection/injury, situational factors), the present results suggested that people over 65 years old most fear the stimuli related to the natural environment, followed by animals, blood/injection/injuries, and specific situations. As discussed above, we do not know of previous studies that have reported the possible differences among the rates of different types of phobias by gender and age. The present study observed that women are up to seven times more likely to suffer from an animal-specific phobia than men and twice as likely to suffer from a specific phobia related to the natural environment than men. Although tentative, these results might be explained by the cultural aspects and related to the traditional gender roles assigned to women that encourage a greater societal permissiveness and the ability to express their fears and emotions. Women express their emotions more than men and are more likely to turn to mental health services when they have an anxiety problem [55]. Women use mental health services 50% more than men [55].

With regard to age, the 12-month prevalence of all types of specific phobias decreased (significant values were found in the case of phobias of the natural environment).

The current study revealed that, along with age and gender, social support (expressed as a greater number of significant people) should be considered as a protective factor against phobias among this age group.

The mean age of onset of specific phobia was 38.78 (sd = 21.61) years, and the average recency age was 57.5 years. Thus, the mean duration of the phobia was approximately 20 (sd = 20) years; no significant differences were found between men and women with regard to the duration of the phobia. When analyzing the trends of this disorder, the first thing that stands out is the negative correlation between age of onset and duration of disorder (r = 0.69), which might indicate that the onset of this disorder in younger people corresponds to a greater duration. A closer analysis identified two groups: one that had a single episode of specific phobia throughout the lifespan with a relatively early onset and another group that suffered from an initial episode of specific phobia at an older age and still lives with this condition. These data suggest that people with specific phobia disorder could carry this problem throughout their lives if more adapted and effective detection/intervention programs are not established.

The results indicated that the situation that generated the most anxiety among people with specific phobia disorder was being in a crowd or in a line of people, whereas the least was being in a public place (e.g., a store, market, theater, or parking lot). Significant differences were found between men and women with regard to the fear of “traveling alone or taking a long trip”, where women were four times more likely to be anxious than men. Again, these data can be explained by gender differences: women are at a greater risk of physical aggression than men when they go alone; thus, women develop this fear to a greater extent. Furthermore, the traditional gender role of women imbues them with more passivity and less personal autonomy, which might help explain the differences in this particular situation. However, no significant differences were observed based on age with regard to any of the problematic situations studied.

The results of this study indicate that living things, such as insects, snakes, birds, or other animals; heights; enclosed spaces, such as a caves, tunnels, or elevators; and going to the dentist or the hospital generated the greatest anxiety in the total sample. Women are more likely to be afraid of living things, such as insects, snakes, birds, or other animals; thunderstorms, thunder, or lightning; being in calm waters, such as a swimming pool or lake; and enclosed spaces. such as caves, tunnels, or elevators. People were less likely to have these fears with age. These findings refute the common thought that people become more fearful with age [56]. 

The results of this study indicate that of the people with specific phobia over the last year, the most frequent symptoms were a stronger or faster heartbeat, dry mouth, shortness of breath, and feeling suffocated. No significant differences were found between men and women or by age group.

Having specific phobia disorder decreased the level of functioning and negatively affected the quality of life of people over 65 years old, according to the three measures used (the WHOQOL-BREF, the overall WHODAS II, and the HoNOS65+). These data corroborate those of previous studies concerning the effects of anxiety [15,38,39,40,57] and in particular the specific phobia disorders [31,32,37,38,39,40] on the level of functioning and quality of life of elderly people. The restricted lifestyle resulting from fear and avoidance in specific phobia is likely to contribute to functional impairment [31]. Observed functional impairment in specific phobia can be partly explained by high cooccurrence with other disorders [58]. 

On the other hand, this study has certain limitations. One has to do with the representativeness of the sample. In the present study, exclusion criteria were applied for various technical reasons, barring people with severe cognitive deficit or who could not be interviewed due to some sort of cognitive deficit or being nursing home residents, homeless, non-Spanish speakers, or people over 85 years old. The sample size and sampling method do not allow us to generalize to the entire elderly population of the Community of Madrid, but it does provide a broad view of the situation. Second, all information about lifetime prevalence was reported retrospectively. This could have led to recall bias, which has been suggested to lead to underestimated lifetime prevalence rates of common mental disorders [59]. Third, the results are based on DSM-IV-TR criteria for specific phobia and using DSM-5 diagnoses could have led to different results. Given the fact that the core features have remained the same, strongly differing prevalence estimations would not be expected.

The results suggest that specific phobia is associated with a considerable decline in the level of functioning and quality of life of people over 65, which can become serious and require support to ensure elderly people’s sustained autonomous functioning and full social participation. These findings suggest that specific phobia deserves attention of clinicians and researchers in view of its direct effects on the level of functioning and quality of life of people over 65. Ultimately, these data suggest the need for primary healthcare professionals to include the detection of specific phobia disorders in their protocols because people do not receive treatment for this problem and they might carry it throughout their lives. We found that the mean duration of specific phobia disorder was 19.73 years, which decreases the level of functioning and worsens the quality of life of people over 65 years old.

## 5. Conclusions

The results show a 12-month prevalence of specific phobia disorder of approximately 12% among people over 65 years old. This study found a higher prevalence than all other previous studies of this age group. The use of adapted diagnostic tools and age-adjusted sampling systems might be the basis of this difference. We found that women were more than twice as likely to suffer from a specific phobia disorder than men; furthermore, its prevalence decreased in both genders with age. These results highlight the importance of considering gender and age when studying specific anxiety disorders in people over 65 years old. Likewise, these differences are relevant to the identification of disorders and the personalization of treatments offered to this population in healthcare services. This study reveals for the first time the differences among the rates of different types of phobias by gender and age. The present results suggested that people over 65 years old most fear the stimuli related to the natural environment, followed by animals, blood/injection/injuries, and specific situations. Women are up to seven times more likely to suffer from an animal-specific phobia than men and twice as likely to suffer from a specific phobia related to the natural environment than men. These results might be explained by the cultural aspects and related to the traditional gender roles assigned to women that encourage a greater societal permissiveness and the ability to express their fears and emotions. The current study revealed that, along with age and gender, social support should be considered as a protective factor against phobias among this age group. The mean duration of the phobia was approximately 20 years. These data suggest that people with specific phobia disorder could carry this problem throughout their lives if more adapted and effective detection/intervention programs are not established. Having specific phobia disorder decreased the level of functioning and negatively affected the quality of life of people over 65 years old. Our study adds significantly to the literature by suggesting the need for primary healthcare professionals to include the detection of specific phobia disorders in their protocols because people do not receive treatment for this problem and they might carry it throughout their lives.

## Figures and Tables

**Table 1:** Sociodemographic characteristics of the sample (N = 555).

Socio-demographic Characteristics	Nº	Prevalence (%)
**Gender**		
Men	267	48.1
Women	288	51.9
**Age (Average)**		
65–74	296	53.3
75–84	259	46.7
**Country born**		
Spain	547	98.6
Other	8	1.4
**Parents born in the same country**		
No	11	2
Yes	544	98
**Marital Status**		
Married	336	60.5
Separated	13	2.3
Divorced	28	5
Widower	151	27.2
Never been married	26	4.7
Other	1	0.2
Widower since (nº ages)	13.09 (0–50)	
**School/education**		
No	258	46.5
Yes	297	53.5
**Years of schooling**		
0–3	88	15.9
4–12	338	61.1
13+	127	23
**Work status**		
Retired	400	72.1
Homemaker/housewife	137	24.7
Working/employed	13	2.3
Unemployed	4	0.7
Other	1	0.2

**Table 2:** Twelve-month prevalence rates of specific phobia disorder (gender and age differences) and prevalence rates (vital, 12 months, and current) by type of specific phobia disorder (animals, natural environments, blood/injection/injury, and situational).

			Prevalence Rate					Odds Ratio	
Disorder	N(555)	INE 2011	Total	Men	Woman	65–74	75–84	Men/woman	65–74/75–84
Specific phobia (12 months)	67	111438	0.13	0.07	0.17	0.16	0.08	0.38 *** (0.21–0.69)	2.08 ** (1.18–3.79)
**Type of specific phobia**									
Animals (vital)	43	73922	0.08	0.02	0.13	0.10	0.05	0.13 *** (0.04–0.33)	1.9 (0.95–3.98)
Animals (12 months)	23	39701	0.05	0.01	0.07	0.05	0.03	0.1 *** (0.01–0.4)	1.38 (0.54–3.68)
Animals (month)	14	24163	0.03	0.00	0.05	0.03	0.02	0.08 ** (0–0.54)	1.59 (0.47–6.13)
Natural environment (vital)	45	75145	0.09	0.06	0.10	0.11	0.05	0.51 * (0.25–1.01)	2.29 * (1.14–4.87)
Natural environment (12 months)	27	45381	0.05	0.03	0.07	0.07	0.03	0.36 * (0.13–0.91)	2.6 * (1.04–7.42)
Natural environment (month)	15	24880	0.03	0.02	0.03	0.04	0.02	0.53 (0.14–1.73)	2.46 (0.72–10.71)
Blood-injections-injuries (vital)	31	49672	0.06	0.06	0.06	0.06	0.05	1.01 (0.46–2.24)	1.41 (0.64–3.26)
Blood-injections-injuries (12 months)	19	30778	0.04	0.03	0.04	0.04	0.03	0.62 (0.2–1.74)	1.21 (0.44–3.53)
Blood-injections-injuries (month)	7	10216	0.01	0.02	0.01	0.01	0.02	2.72 (0.44–28.84)	0.65 (0.09–3.9)
Situational (vital)	33	54871	0.06	0.04	0.07	0.07	0.05	0.6 (0.26–1.31)	1.57 (0.72–3.58)
Situational (12 months)	14	23490	0.03	0.01	0.03	0.02	0.03	0.42 (0.1–1.49)	0.65 (0.18–2.17)
Situational (month)	6	10207	0.01	0.01	0.01	0.01	0.01	0.54 (0.05–3.78)	0.87 (0.12–6.58)

**Note:** *** *p* < 0.001. ** *p* < 0.01. * *p* < 0.05.

**Table 3:** Logistic regression for psychosocial factors: age, gender, and number of significant people. Odds ratio (OR).

Psychosocial Factors	Coefficient	std. Error	OR	CI (95%) of OR
**Age**	−0.07	0.02	0.93	(0.89–0.97)
**Gender (men/women)**	−0.93	0.29	0.40	(0.22–0.69)
**Number of significant people**	−0.05	0.02	0.95	(0.91–0.98)

**Table 4:** Description of the symptoms of the specific phobia disorder in the Community of Madrid sample (problematic situations, phobic stimuli, and general symptomatology). Prevalence rate, 12 months, odds ratio for gender, age, and contrasts (OR = 1).

			Prevalence Rate					Odds Ratio	
Variable	N (555)	Statistics National Institute (INE) 2011	Total	Hombres	Women	65–74	75–84	Men/Women	65–74/75–84
**Problematic situations**									
Being outside home alone	24	39690	0.27	0.21	0.30	0.21	0.35	0.63 (0.18–1.95)	0.5 (0.17–1.45)
Travelling on a bus. train. underground/subway or using other public transport	24	40287	0.27	0.24	0.28	0.29	0.24	0.83 (0.25–2.49)	1.3 (0.44–4.03)
Being in a crowd or standing in line	43	69131	0.47	0.52	0.46	0.46	0.50	1.26 (0.47–3.37)	0.87 (0.34–2.22)
Being in a public place. such as shops. markets. department store. a theatre. or car park	7	12171	0.08	0.00	0.11	0.07	0.09	0 (0–1.4)	0.8 (0.13–5.8)
Travelling alone or going on a long trip	17	28939	0.20	0.07	0.25	0.14	0.26	0.23 * (0.02–1.11)	0.47 (0.14–1.55)
Crossing a bridge	29	48386	0.33	0.28	0.34	0.34	0.29	0.73 (0.24–2.09)	1.23 (0.45–3.5)
**Stimuli**									
Living things like insects. snakes. birds. or other animals	143	240091	0.27	0.13	0.38	0.31	0.20	0.25 *** (0.16–0.39)	1.84 ** (1.22–2.79)
The sight of blood	50	80904	0.09	0.09	0.09	0.11	0.07	1.09 (0.58–2.03)	1.62 (0.86–3.15)
Getting an injection	22	35917	0.04	0.04	0.04	0.05	0.02	0.9 (0.34–2.3)	2.41 (0.88–7.63)
Going to the dentist or hospital	61	99273	0.11	0.09	0.13	0.12	0.10	0.72 (0.4–1.28)	1.2 (0.68–2.15)
Heights	104	167760	0.19	0.19	0.19	0.20	0.17	1 (0.64–1.56)	1.18 (0.75–1.87)
Storms. thunder. or lightning	53	89110	0.10	0.04	0.15	0.12	0.07	0.25 *** (0.11–0.51)	1.97 * (1.05–3.84)
Being in water. such as a lake or swimming pool	21	35095	0.04	0.01	0.06	0.04	0.03	0.24 ** (0.06–0.76)	1.17 (0.45–3.21)
Flying in an airplane	51	82993	0.09	0.08	0.10	0.11	0.07	0.8 (0.43–1.49)	1.68 (0.89–3.25)
Being in a closed space like a basement. tunnel. or elevator	86	144121	0.16	0.09	0.22	0.18	0.13	0.36 *** (0.21–0.61)	1.41 (0.86–2.33)
Any other situations	8	13353	0.02	0.00	0.02	0.01	0.02	0.15 * (0–1.19)	0.87 (0.16–4.74)
Without fear	285	433604	0.50	0.61	0.43	0.45	0.59	2.07 *** (1.45–2.95)	0.56 *** (0.39–0.79)
**General Symptomatology**									
Pounding or racing heart	38	62499	0.42	0.45	0.41	0.43	0.41	1.17 (0.43–3.12)	1.07 (0.42–2.8)
Sweating	24	39059	0.26	0.31	0.25	0.30	0.21	1.37 (0.45–4.04)	1.67 (0.56–5.45)
Trembling or shaking	16	25575	0.17	0.24	0.15	0.16	0.21	1.83 (0.51–6.33)	0.74 (0.22–2.63)
Dry mouth	29	47157	0.32	0.34	0.31	0.36	0.26	1.16 (0.4–3.25)	1.54 (0.56–4.5)
Difficulty breathing or shortness of breath	28	45547	0.31	0.34	0.30	0.30	0.32	1.25 (0.43–3.53)	0.91 (0.33–2.56)
Sensation of choking	26	42178	0.29	0.41	0.23	0.32	0.24	2.35 (0.82–6.77)	1.53 (0.53–4.71)
Pain or discomfort in chest	5	8044	0.05	0.03	0.07	0.07	0.03	0.51 (0.01–5.5)	2.52 (0.24–128.66)
Stomach pains or discomfort in stomach	9	15305	0.10	0.07	0.11	0.13	0.06	0.57 (0.05–3.3)	2.27 (0.4–23.71)
Feeling dizzy or lightheaded	14	23452	0.16	0.21	0.13	0.20	0.09	1.72 (0.44–6.4)	2.5 (0.59–15.11)
Things around you seemed unreal	9	14474	0.10	0.17	0.07	0.13	0.06	2.93 (0.58–16.12)	2.27 (0.4–23.71)
Afraid of losing control or acting crazy	10	16015	0.11	0.21	0.07	0.16	0.03	3.66 (0.78–19.33)	6.22 (0.8–284.83)
Afraid of dying	15	23579	0.16	0.28	0.11	0.20	0.12	2.9 (0.81–10.73)	1.82 (0.48–8.59)
Hot flushes or chills	18	29367	0.20	0.24	0.18	0.23	0.15	1.44 (0.41–4.73)	1.74 (0.51–6.94)
Tingling or numbness in hands. arms. or legs	13	21205	0.14	0.14	0.15	0.13	0.18	0.93 (0.19–3.73)	0.67 (0.17–2.67)
No symptoms	23	37042	0.25	0.21	0.28	0.20	0.35	0.68 (0.19–2.12)	0.45 (0.15–1.32)

**Note:** *** *p* < 0.001, ** *p* < 0.01, * *p* < 0.05.

**Table 5:** Adjusted biserial-point correlations for gender and age (relation between specific phobia disorder and global WHO Disability Assessment Schedule (WHODAS II), total Health of the Nation Outcome Scales for Older Adults (HoNOS65+), and total World Health Organization Quality of Life Brief (WHOQOL-BREF)).

Disorder		R	*p*-Value	R^2^
Specific phobia	Global WHODAS II	0.12	0.004	0.01
Specific phobia	Total HoNOS65+	0.19	0.000	0.03
Specific phobia	Total WHOQOL-BREF	−0.08	0.048	0.01
